# Rapid Rather than Gradual Weight Reduction Impairs Hemorheological Parameters of Taekwondo Athletes through Reduction in RBC-NOS Activation

**DOI:** 10.1371/journal.pone.0123767

**Published:** 2015-04-14

**Authors:** Woo Hwi Yang, Oliver Heine, Sebastian Pauly, Pilsang Kim, Wilhelm Bloch, Joachim Mester, Marijke Grau

**Affiliations:** 1 Department of Molecular and Cellular Sport Medicine, Institute of Cardiovascular Research and Sport Medicine, German Sport University Cologne, Cologne, Germany; 2 Institute of Training Science and Sport Informatics, German Sport University Cologne, Cologne, Germany; 3 German Research Centre for Elite Sports, German Sport University, Cologne, Germany; 4 Olympic Training Centre Rhineland, Cologne, Germany; Massachusetts Institute Of Technology, UNITED STATES

## Abstract

**Purpose:**

Rapid weight reduction is part of the pre-competition routine and has been shown to negatively affect psychological and physiological performance of Taekwondo (TKD) athletes. This is caused by a reduction of the body water and an electrolyte imbalance. So far, it is unknown whether weight reduction also affects hemorheological properties and hemorheology-influencing nitric oxide (NO) signaling, important for oxygen supply to the muscles and organs.

**Methods:**

For this purpose, ten male TKD athletes reduced their body weight by 5% within four days (rapid weight reduction, RWR). After a recovery phase, athletes reduced body weight by 5% within four weeks (gradual weight reduction, GWR). Each intervention was preceded by two baseline measurements and followed by a simulated competition. Basal blood parameters (red blood cell (RBC) count, hemoglobin concentration, hematocrit, mean corpuscular volume, mean cellular hemoglobin and mean cellular hemoglobin concentration), RBC-NO synthase activation, RBC nitrite as marker for NO synthesis, RBC deformability and aggregation parameters were determined on a total of eight investigation days.

**Results:**

Basal blood parameters were not affected by the two interventions. In contrast to GWR, RWR decreased activation of RBC-NO synthase, RBC nitrite, respective NO concentration and RBC deformability. Additionally, RWR increased RBC aggregation and disaggregation threshold.

**Conclusion:**

The results point out that a rapid weight reduction negatively affects hemorheological parameters and NO signaling in RBC which might limit performance capacity. Thus, GWR should be preferred to achieve the desired weight prior to a competition to avoid these negative effects.

## Introduction

In combat sports such as Taekwondo (TKD), body weight reduction belongs to the pre-competition routine. Athletes must qualify for a competitive weight category of different weight classes by weigh-in the day before the official competition. Although experts recommend losing weight by gradual weight reduction (GWR) in order to sustain performance capacity [1,2], rapid weight reduction (RWR) protocols are commonly used. RWR is characterized by temporary weight loss that allows the athletes to fight at the upper limit of the next lighter weight category in order to fight against smaller and weaker opponents [3]. RWR is achieved by fluid and food restriction, increased exercise intensity and volume with thermal clothing, sauna sessions and dehydration [2–4]. These methods are known to negatively affect athlete`s health and exercise performance. RWR increases heart rate, impairs thermoregulatory processes and decreases plasma volume [1]. Acute dehydration during RWR further increases hematocrit (Hct) and thus blood viscosity which may impair oxygen (O_2_) transport to the muscle cells and muscular performance [5–7].

In this regard, deformability and aggregation of red blood cells (RBC) as main properties of hemorheology are determining factors of the flow resistance. Aggregation is described as face-to-face rouleaux formation and affected by the presence of plasma macromolecules and fibrinogen [8]. RBC aggregation is responsible for the shear thinning behavior and can influence hemodynamics, RBC distribution and flow dynamics in the microcirculation. Increased RBC aggregation can decrease the tissue perfusion that negatively affects performance capacity of athletes [7,9–11]. RBC deformability is determined by internal fluidity, cell surface area-to-volume ratio and physical properties of the membrane and cytoskeleton [12,13]. RBC deformability is also responsible for the shear thinning behavior and a decline in RBC deformability impedes and reduces the blood flow through the capillaries [14,15]. RBC deformability has been shown to be affected by nitric oxide (NO) which, in red blood cells (RBC), is produced by RBC-NO synthase (RBC-NOS) [16,17]. RBC-NOS activity thus could contribute to the oxygen supply of the muscles and organs through regulation of the microcirculation [18–20].

So far, it is unknown how RWR and GWR affect NO production and subsequent hemorheological properties. Based on our previous findings of reduced performance capacity after RWR [2], we hypothesize that the hemorheological parameters of TKD athletes were impaired after RWR but not GWR which is associated to a reduction in RBC-NOS activation and NO production.

## Materials and Methods

### Ethical approval

The protocols used in this study were approved by the ethics committee of the German Sport University Cologne. The applied protocols align with the Declaration of Helsinki and all participants gave written informed consent to participate in this study.

### Subjects

Ten male well-trained TKD athletes of different weight classes (n = 10) participated in this study. Basal anthropometric parameters of the subjects were as follows (Mean ± SD): age 21.1 ± 5.48 years; height: 1.74 ± 0.08 m; weight: 71.6 ± 11.1 kg. They were familiar with TKD specific training and endurance loads for at least 10 years and possessed individual experiences in body weight reduction. Subjects did not take any medication during the entire procedure and abstained from alcohol and nicotine consumption for at least 24 hours prior to the experiment.

### Study design and blood sampling

**Fig 1:**
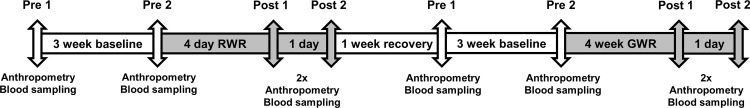
Experimental study design. The study consisted of a three week baseline phase followed by a four day rapid weight reduction (RWR) with a simulated competition day. This was followed by a one week recovery time, a second three week lasting baseline phase, a four week gradual weight reduction (GWR) and a simulated competition day. At a total of eight investigation days (see arrows) anthropometry was measured and venous blood samples were taken.

The study was characterized by two cycles of weight reduction comprising of weight reduction by 5% during four days (RWR) and four weeks (GWR), respectively. Both interventions were separated by a recovery phase and measurements took place on a total of eight investigation days including measurements at baseline (baseline 1 + 2) [21], after weight reduction (post 1) and after re-hydration and ingestion (post 2) (Fig 1).

During RWR, weight loss was achieved by participants’ individually used methods which included higher training intensity/volume and training sessions with thermal clothing, fasting and dehydration. The nutritional- and activity status for each subject was analyzed and the individual demand for GWR was calculated by a nutritionist and exercise physiologist of the Institute of Biochemistry (German Sport University Cologne) [2]. Prior to GWR, anthropometric parameters were restored to ensure equal starting conditions pre RWR and pre GWR. Weight loss during GWR was thus achieved by combining exercise with minimal caloric intake (low-energy and high density foods) (see for details [2]).

All athletes continued their sport specific training schedule (6–8 h per week) with an additional running-training session (1h/week). Weight loss was monitored daily during RWR and weekly during GWR using single frequency bioimpedance analyzer BIA (BC-418 MA Tanita, The Netherlands).

Investigations took place in the morning under fasting conditions on each investigation day. Blood samples were taken from the antecubital vein and anticoagulated using sodium-heparin vacutainer (BD Vacutainer, USA). All blood preparations were carried out immediately after blood sampling.

### Blood parameters

Venous anticoagulated whole blood (50μl) was used to measure the number of RBC [*10^6^/μl], hemoglobin concentration (Hb) [g/dl], hematocrit (Hct) [%], mean corpuscular volume (MCV) [fl], mean cellular hemoglobin (MCH) [pg] and mean cellular hemoglobin concentration (MCHC) [g/dl] using the Sysmex Digitana KX-21N system (Germany).

### Immunohistochemical staining of phosphorylated RBC-NOS^Ser1177^

To determine RBC-NOS phosphorylation at serine 1177 residue, representing activation of the enzyme, anticoagulated RBCs were fixed with 4% paraformaldehyde (v/v; 1/1) immediately after blood sampling and separated as described by Suhr et al. [18] and Grau et al. [19,20]. Fixed RBCs were dispersed on a slide and heat-fixed. Slides were separated into a test and a control area using a grease pencil to ensure an intra-sample control. RBCs were washed with 0.1 M tris-buffered saline (TBS), permeabilized for 30 min in 0.1% trypsin at 37°C, placed in a solution of 2% hydrogen peroxide and 80% methanol/rest H_2_O for 30 min, and treated with 3% milk powder in 0.1 M TBS for 30 min at room temperature (RT). The test area of each slide was incubated with a primary antibody against RBC-NOSSer^1177^ (dilution 1:500, rabbit anti-phospho-eNOSSer^1177^—Millipore, Germany) for 1 hour at RT. The control area was incubated in the absence of the primary antibody. After rinsing with TBS and an incubation step with 3% Normal Goat Serum (Dako, Dekmark), both sections were incubated with the secondary goat-anti-rabbit antibody (dilution 1:400, Dako, Denmark) for 1 hour at RT. A streptavidin-horseradish-peroxidase complex (Sigma-Aldrich, St. Louis, USA) was applied as detection system (dilution 1:400) for 30 min at RT. The staining was developed using 3,3-diaminobenzidine-tetrahydrochloride solution (Sigma-Aldrich, St. Louis, USA) in 0.1 M TBS and after dehydration of the samples in an ascending alcohol series (70%, 96% and 100% ethanol) and xylene, samples were covered using Entellan (Merck/Millipore, Germany) and a cover slide.

For staining intensity detection, pictures of the stained slides were taken using a Leica microscope coupled to a CCD-camera (DXC-1850P, Sony, Germany) and the semi-quantitative analysis of the grey values was conducted using the software “Image J” (National Institutes of Health, USA). Magnification for all images was 400-fold. The edge of red blood cells was marked and the grey values were calculated in arbitrary units. The grey values of a total of 100 red blood cells from at least four pictures were determined in the test area and subtracted from the background value which was measured in a cell-free area of the slide. Then, the grey values of a total of 15 red blood cells from at least two pictures were determined in the control area and also subtracted from the background value. Finally, grey values of RBCs from test and control areas were subtracted to obtain net staining intensities.

### Measurement of RBC nitrite

After blood sampling, RBCs were separated from the plasma by centrifugation at 5,000xg and 4°C for 1 min. RBCs were immediately mixed with a ferricyanide-based preservation solution (1:5-ratio; preservation solution: RBCs) to preserve nitrite in RBCs. The solution consists of 0.8 M ferricyanide, 0.1 M N-ethylmaleimide, and Igepal (10% of total volume of preservation solution) [20,22]. The samples were mixed and snap-frozen in liquid nitrogen and stored at -80°C until measurement. For nitrite measurement in RBCs, methanol (VWR international, Germany) was added to the frozen samples in a 1:2-ratio to remove proteins and centrifuged at 21,000xg and 4°C for 15 min. Nitrite level of the supernatant was determined by injecting 100 μL into an acidified tri-iodide solution that reduces nitrite to NO gas. Along with a helium gas-stream, NO was purged into an ozone-based chemiluminescence NO detector (CLD 88 NO, Ecophysics, Switzerland) [23]. The Power Chrome software (Ecophysics, Switzerland) was used to integrate the area under the curve. All samples were measured in triplicate. Using aqueous calibration solutions with known nitrite concentration allowed calculation of sample nitrite content.

### Measurement of RBC rheological parameters deformability and aggregation

RBC deformability and aggregation were both measured using the Laser-Assisted Optical Rotational Cell Analyzer (LORCA; RR Mechatronics, The Netherlands) as described elsewhere [24]. This system is a combination of an ektacytometer to measure deformability and a photometric system for measuring aggregation. RBCs were separated from other blood cells and plasma by centrifugation at 800xg and 4°C for 10min. Platelets and leukocytes were discarded, RBCs were then reconstituted in autologous plasma and the hematocrit was adjusted to 40%. RBC deformability was determined immediately after blood sampling at various fluid shear stresses by laser diffraction analysis at 37°C. RBCs were mixed with an isotonic viscous medium (0.14 mM Polyvinylpyrrolidone (PVP), osmolality 300 mOsmol*L-1, viscosity 30 mPa*s at 37°C; Mechatronics, The Netherlands) in a 1:250 ratio [25]. The blood/PVP sample was immediately sheared between two coaxial glass cylinders (Couette system) with a laser beam directed through the sheared sample. The diffraction pattern produced by shear stress induced deformed red cells was analyzed and an elongation index (EI) was calculated by the LORCA software on the basis of the vertical and horizontal radius of the diffraction pattern. A total of nine shear stresses between 0.3 and 50 Pa were incrementally applied and a total of 50 measurements were performed for each shear stress and a respective mean value for each shear stress was calculated by the LORCA software. According to Baskurt et al. [26] the Lineweaver-Burke equation was used to calculate the maximum deformability (EI max) on the basis of the EI values obtained for each shear rate.

For aggregation measurements by syllectometry, samples were fully oxygenated with the use of a Roller Mixer (RM5, 36 rpm). Samples were then filled between the two glass cylinders and measurements started at 37°C. The syllectogram (transmitted light intensity versus time) distinguishes four behavioral stages: 1) Disaggregation which was achieved by increasing shear rate to 500s^-1^ for 3 sec 2) RBC-shape recovery upon stop of motor, 3) Rouleaux formation immediately followed by 4) 3D aggregate formation during the following 120 sec of aggregation measurement. Thus, the laser beam is directed through the sample and backscattered with the reflected light being recorded by two photodiodes built into the inner stationary cylinder.

The LORCA software then calculated the aggregation index (AI, %) which depends on the extent and kinetics of aggregation. A following iteration procedure was performed to determine the threshold shear rate balancing RBC aggregation and disaggregation. The calculated dIsc min is defined as the minimum change in backscatter intensity found during the iteration procedure enabling disaggregation with γ at dIsc min (1/sec) being the shear rate at which dIsc min was found.

### Statistical Analysis

Statistical software packages Origin 8.5 Pro (Northampton, USA) and GraphPadPrism 6 (La Jolla, USA) were used for statistical analyses of the data and graphical representation of data.

Data were analyzed by a two-way ANOVA with Fisher post-hoc tests (protocol by time). The data are presented as mean ± standard error of means (S.E.M.) unless described otherwise. Statistical differences were considered to be significant for values of *P*<0.05, *P*<0.01 and *P*<0.001.

## Results

### Anthropometric data

The body weight was reduced by 5% in Post 1 of RWR and GWR, respectively. Body weight, significantly increased at Post 2 after RWR and GWR, respectively (*P*<0.001). The increase in Post 2 for GWR was significantly lower than in Post 2 of RWR (*P*<0.001). The body water was significantly reduced after RWR (*P*<0.001) and GWR (*P*<0.01) but significantly increased again between Post 1 and Post 2, respectively (RWR: *P*<0.001, GWR: *P*<0.05). Significant higher body water was found in Post 1 of GWR compared to Post 1 of RWR as well as in Post 2 of RWR compared to Post 2 of GWR (*P*<0.05). Differences in body fat were only observed in GWR. Values significantly decreased in Post 1 compared to Pre 1 and 2 (*P*<0.01). Also, a significant difference in Post 1 of GWR compared to Post 1 of RWR was observed (*P*<0.01) (Table 1).

**Table 1:** Anthropometric data measured using bioimpedance analyzer BIA (body weight, body water and body fat).

	RWR				GWR			
	Pre 1	Pre 2	Post 1	Post 2	Pre 1	Pre 2	Post 1	Post 2
Body weight (kg)	71.6 ± 11.1	71.7 ± 11.5	68.0 ± 10.5^a^ ** ^,^ ^b^ **	70.8 ± 10.8^c^ **	71.7 ± 11.1	71.1 ± 11.1	67.7 ± 10.6^e^ ** ^,^ ^f^ **	68.9 ± 10.6^g^ ** ^,^ ^d^ **
Body water (kg)	45.0 ± 5.0	45.3 ± 5.2	43.3 ± 4.8^a^ ** ^,^ ^b^ **	45.4 ± 4.8^c^ **	45.6 ± 5.3	45.0 ± 5.2	44.2 ± 5.0^e^ ** ^,c^ *	44.7 ± 4.8^g^ * ^,^ ^d^ *
Body fat (%) BIA	13.3 ± 4.6	12.9 ± 4.7	12.2 ± 4.8	11.7 ± 4.8	12.7 ± 4.1	13.0 ± 4.3	10.0 ± 5.3^e^ ** ^,^ ^f^ ** ^,c^ **	10.6 ± 5.1

Data are present as mean ± S.D. of n = 10.

RWR: rapid weight reduction; GWR: gradual weight reduction; Procedure main effect: compared to

a: pre 1 RWR,

b: pre 2 RWR,

c: post 1 RWR,

d: post 2 RWR,

e: pre 1 GWR,

f: pre 2 GWR,

g: post 1 GWR;

*P<0.05,

** *P*<0.01.

### Blood parameters

The measured blood parameters including RBC number, Hb, Hct, MCV, MCH and MCHC showed no significant differences during or between RWR and GWR (Table 2).

**Table 2:** Hematological data of all subjects.

	RWR				GWR			
	Pre 1	Pre 2	Post 1	Post 2	Pre 1	Pre 2	Post 1	Post 2
RBC (*10^6^/μl)	5.26 ± 0.17	5.14 ± 0.23	5.25 ± 0.28	4.87 ± 0.34	5.02 ± 0.18	5.24 ± 0.35	5.13 ± 0.40	5.03 ± 0.34
Hb (g/dl)	15.29 ± 1.09	14.92 ±1.44	15.25 ± 1.27	14.58 ± 2.23	14.59 ± 1.11	15.19 ± 1.52	14.87 ± 1.38	14.43 ± 1.33
Hct (%)	45.65 ± 2.30	44.56 ± 3.30	45.75 ± 2.73	43.03 ± 3.42	43.83 ± 2.19	45.77 ± 3.81	44.58 ± 3.10	43.54 ± 3.08
MCV (fl)	86.92 ± 4.87	86.76 ± 4.80	87.16 ± 4.37	87.05 ± 4.86	87.36 ± 4.73	87.38 ± 5.08	87.16 ± 4.92	86.63 ± 4.90
MCH (pg)	29.12 ± 2.29	29.05 ± 2.35	29.07 ± 2.40	29.31 ± 2.27	29.10 ± 2.44	28.99 ± 2.17	29.07 ± 2.24	28.72 ± 2.35
MCHC (g/dl)	33.46 ± 1.15	33.43 ± 1.22	33.31 ± 1.27	33.63 ± 1.15	33.26 ± 1.34	33.16 ± 1.06	33.32 ± 1.17	33.11 ± 1.24

Data are present as mean ± S.D. of n = 10.

RBC: red blood cell; Hb: hemoglobin; Hct: hematocrit; MCV: mean corpuscular volume; MCH: mean corpuscular hemoglobin; MCHC: mean corpuscular hemoglobin concentration; RWR: rapid weight reduction; GWR: gradual weight reduction.

### RBC-NOS^Ser1177^ signal

**Fig 2:**
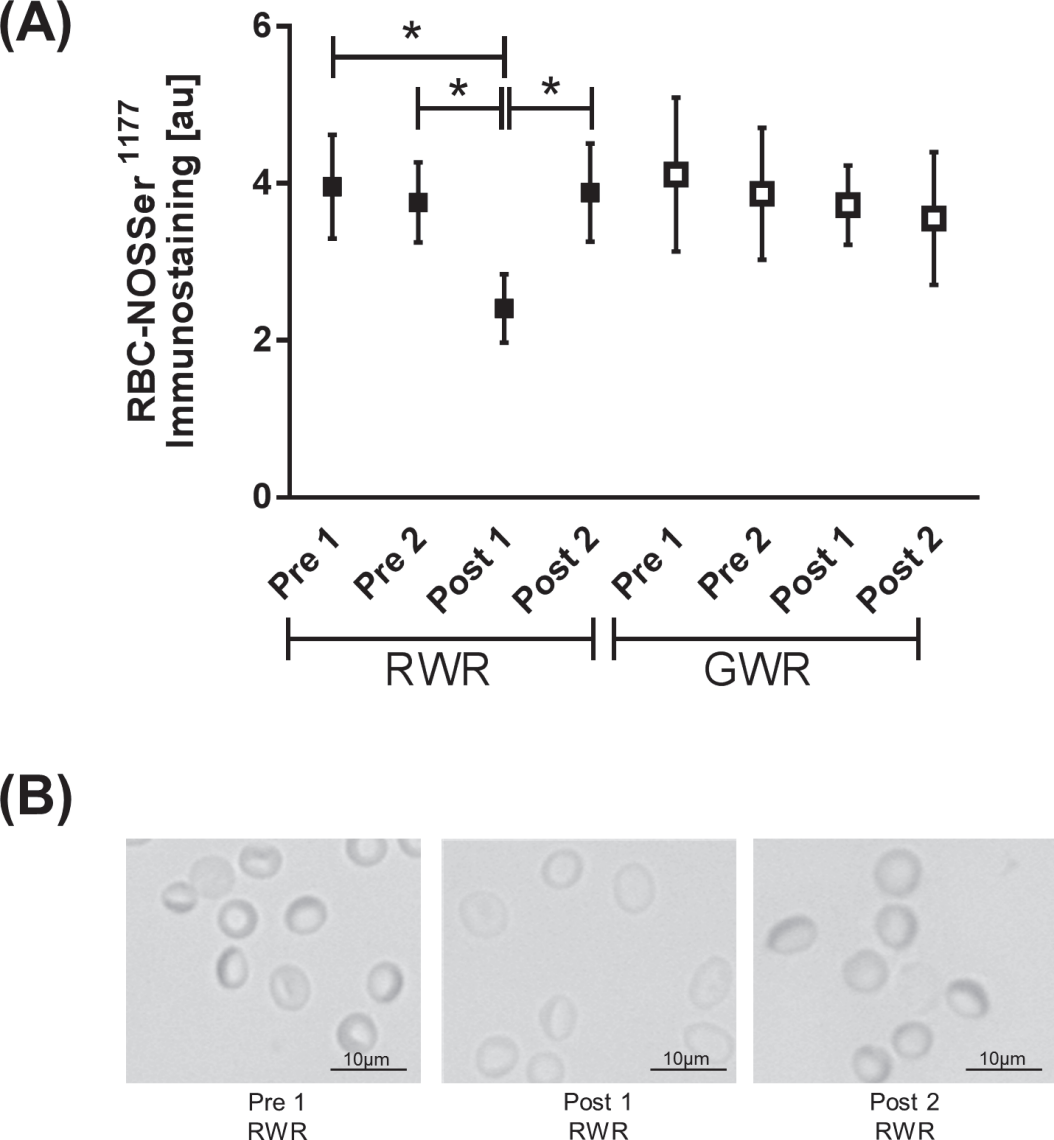
RBC-NOSSer1177 levels during weight reduction protocols. (A) Bars showed statistically decreased RBC-NOS^Ser1177^ phosphorylation Post 1 RWR compared to Pre 1 and 2 RWR (*P*<0.05, respectively). Phosphorylation significantly increased Post 2 RWR compared to Post 1 (*P*<0.05). (B) Pictures show representative RBC-NOS^Ser1177^ photographs of Pre 1, Post 1 and Post 2 of RWR, respectively. Magnification for all images was 400-fold. Data in (A) are presented as mean ± S.E.M (n = 10).

The statistical analysis of gray value against total RBC-NOS^Ser1177^ obtained during RWR revealed significantly decreased phosphorylation of the enzyme Post 1 compared to Pre 1 and Pre 2 (*P*<0.05), respectively. Phosphorylation significantly increased between Post 1 and Post 2 of RWR (*P*<0.05). No statistical differences were observed during GWR (Fig 2A and 2B).

### RBC nitrite

**Fig 3:**
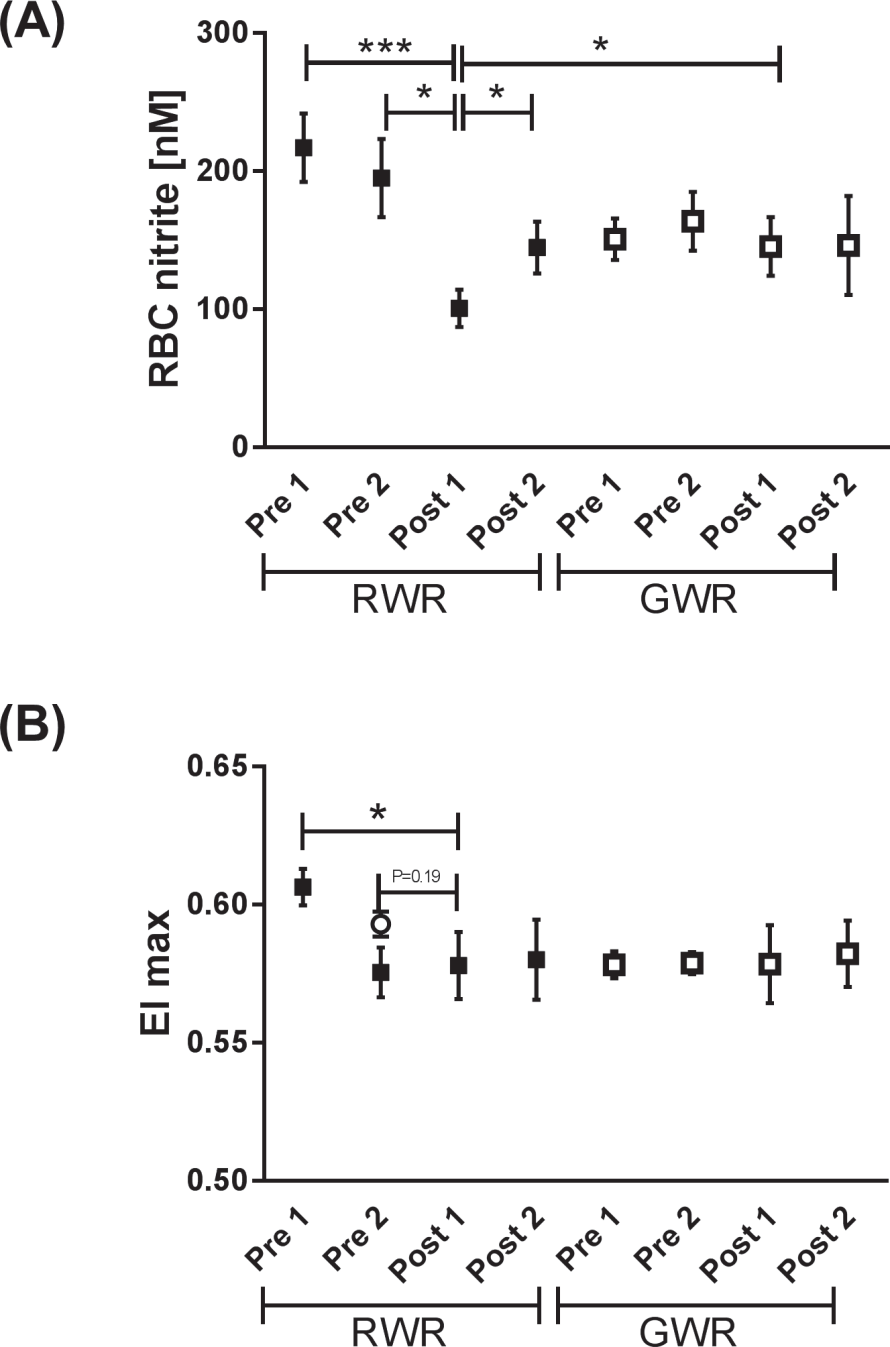
RBC nitrite concentration and maximum deformability (EI max) during weight reduction protocols. (A) During RWR, RBC nitrite significantly decreased from Pre 1 and Post 2 to Post 1 (*P*<0.01 and *P*<0.05, respectively) and increased from Post 1 to Post 2 (*P*<0.05). Nitrite concentration was not affected by GWR but nitrite level Post 1 GWR was significantly higher compared to Post 1 RWR (*P*<0.05). (B) EI max significantly decreased Post 1 compared to Pre 1 of RWR (*P*<0.05). EI max was not affected by GWR. Data in (A and B) are presented as mean ± S.E.M (n = 10).

During RWR, RBC nitrite concentration significantly decreased Post 1 compared to Pre 2 (*P*<0.05) and Pre 1 (*P*<0.001) and increased from Post 1 to Post 2 (*P*<0.05). During GWR, RBC nitrite concentration showed no significant differences. Nitrite levels measured Post 1 during GWR was significantly higher compared to Post 1 RWR (*P*<0.05) (Fig 3A).

### RBC Deformability

Calculated EI max significantly decreased Post 1 compared to Pre 1 (*P*<0.05) during RWR. Values measured during GWR showed no significant differences (Fig 3B).

### RBC Aggregation

**Fig 4:**
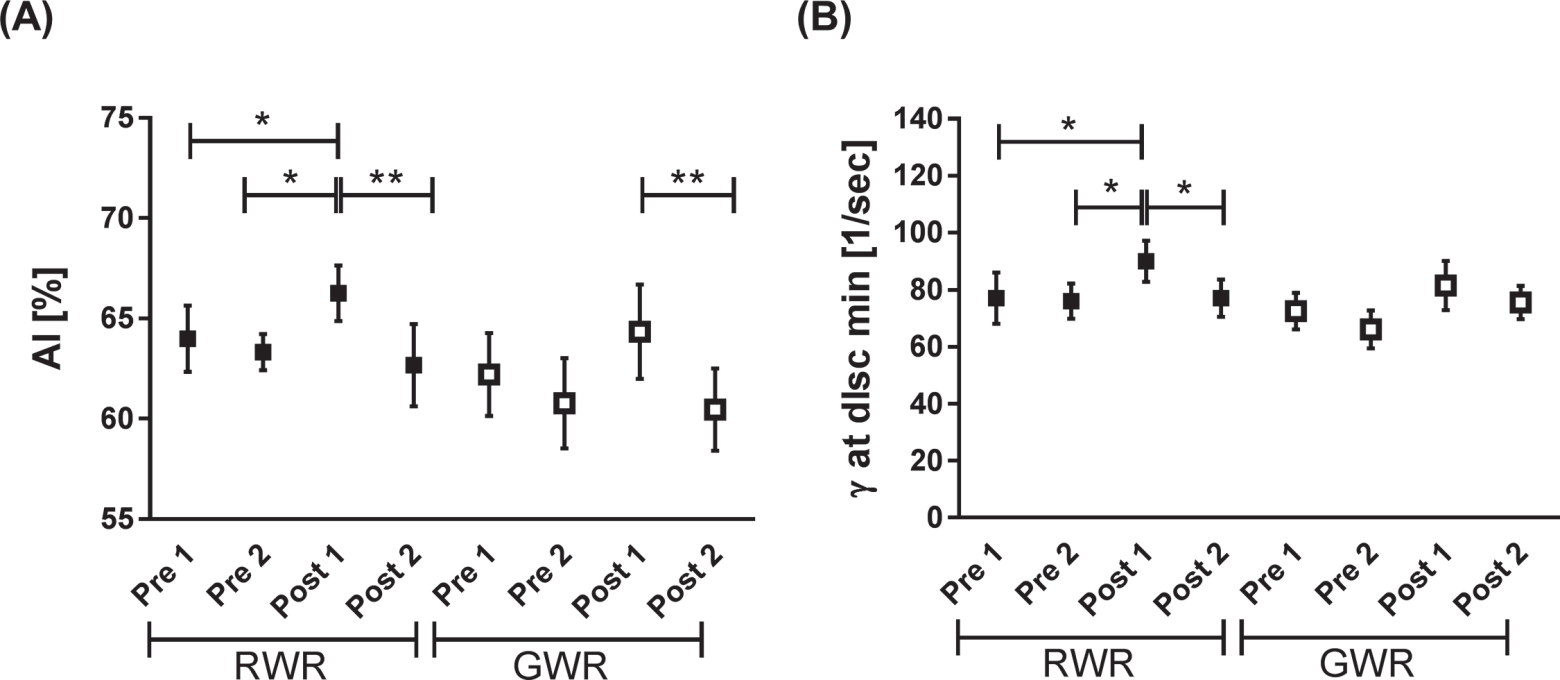
Aggregation index (AI, %) and disaggregation threshold (γ at dISCmin, 1/sec) during weight reduction protocols. (A) During RWR, the aggregation index significantly increased Post 1 compared to Pre 1 and Pre 2 (*P*<0.05, respectively) and decreased again Post 2 (*P*<0.01). During GWR, AI decreased Post2 compared to Post 1 (*P*<0.01). (B) Disaggregation threshold increased Post 1 RWR compared to Pre 1 and Pre 2 RWR (*P*<0.05, respectively) and decreased Post 2 compared to Post 1 (*P*<0.05). No change in disaggregation threshold was measured during GWR. Data in (A and B) are presented as mean ± S.E.M (n = 10).

During RWR, AI (%) significantly increased from Pre 1 and Pre 2 to Post 1 (*P*<0.05, respectively) and decreased from Post 1 to Post 2 (*P*<0.01). During GWR, AI (%) decreased Post 2 compared to Post 1 (*P*<0.01) (Fig 4A). The disaggregation threshold increased during RWR form Pre 1 and Pre 2 to Post 1 (*P*<0.05, respectively) and significantly decreased from Post 1 to Post 2 (*P*<0.05). No significant differences were observed during GWR (Fig 4B).

## Discussion

Our previous findings indicated reduced performance capacity of TKD athletes by RWR [2]. Regarding thereto, the present study was examined to address the question whether the decline in performance capacity may be ascribed to hemorheological alterations, a reduction in RBC-NOS activation and reduced nitrite, respective NO production, during weight loss. The results of this study showed impaired RBC-NOS activation, reduced nitrite content and thus reduced NO production and decreased deformability, coincident with increased aggregation after rapid weight reduction.

Hematological parameters like RBC count, hematocrit, hemoglobin concentration, MCV, MCH and MCHC were investigated as, e.g. MCHC has been shown to sensitively reflect cell viscosity within RBC [27] and increased blood hematocrit was shown to negatively affect blood viscosity. Changes in these parameters were shown to negatively affect performance capacity of athletes [7,9–11] because increased viscosity reduces peripheral blood flow and cardiac output. High hematocrit-levels of athletes commonly occurred after loss of body water during overtraining and/or iron deficiency [28]. The results obtained herein showed that none of the investigated hematological parameters were affected by RWR or GWR. The level of hematocrit showed no alteration although the body water was significantly decreased after RWR [2] but several studies confirmed that the hematocrit does not necessarily increase during weight reduction [29–31].

RBC-NOS activation was determined by measuring phosphorylation at its serine 1177 residue. RBC-NOS ^Ser1177^ decreased during RWR but not during GWR, which indicates decreased enzyme activation [19,32] after rapid weight reduction. This deterioration of its activation was possibly caused by reduced calcium level occurring during fasting and rapid dehydration [2,33,34]. Intracellular calcium and its binding to calmodulin are essential for NOS activity [35,36]. RBC nitrite levels also decreased during RWR. Comparison of caloric intake and composition of food revealed that at baseline 12.3 ± 13.4% of the calories originated from mixed vegetables which are rich in nitrate and nitrite [37] but during RWR only 6.4 ± 8.9% of the calories originated from mixed vegetables. Thus, it is speculated that the reduced nitrate/nitrite uptake may in part explain the reduced nitrite concentration measured in RBC. But reduced RBC nitrite may also be caused by reduced RBC-NOS activation. Studies showed that nitrite sensitively reflects NOS dependent NO production [38,39] and thus reduction in RBC-NOS activation during RWR may account for reduced nitrite, respective NO content in RBC. A decrease in circulating RBC NO has been shown to negatively affect RBC deformability [18,19,40]. In the present study, measurement of RBC deformability revealed differences between the baseline values Pre 1 and Pre 2 of RWR. Six out of ten tested subjects absolved an intensive training camp and participated in the national and international Taekwondo Championships between the two investigation days and the values obtained from these subjects led to the decrease in EI max between Pre 1 and Pre 2 of RWR. No difference between Pre 1 and Pre 2 were measured when excluding these values from the calculation (see symbol in Fig 3B: open circle). The results thus showed a decrease in RBC deformability after RWR which could be ascribed to the reduced RBC-NOS-generated NO. A correlation between these two parameters has recently been shown [20]. Reduced deformability may also be caused by increased mean cellular volume [41] but no changes in MCV were observed during the both interventions.

Our previous study showed decreased glucose levels during RWR [2]. Glucose consumption and decreased ATP (adenosine triphosphate) levels impair rheological properties such as deformability [42–46]. Decreased glucose levels thus may also have contributed to the reduction in RBC deformability in the present study. Further, food restriction may compromise antioxidant mechanisms which under normal conditions guard RBC from the exercise stress as well as lipid peroxidation of the RBC membrane [47,48]. Food restriction may thus have increased oxidative/nitrosative stress [48]. Also, the training intensity of the subjects was increased within the four days of RWR causing, primarily, loss of body water [2]. It was suggested that the intensive exercise sessions may have also increased oxidative/nitrosative stress which reduced NO bioavailability and favored the decrease in RBC deformability as previously observed by others [49–51].

Another hemorheological parameter measured in this study was RBC aggregation. RBCs have a tendency to form aggregates but RBC aggregation is a reversible process. They will separate when external forces such as blood flow, increases [52]. Oxidative stress, which occurs during and after exercise [12,53] was associated with increased RBC aggregation and increased RBC aggregate strength [54–56]. The data presented here proved increased RBC aggregation and aggregation strength during RWR but not GWR in which these parameters only tended to increase. Dehydration which occurred during RWR, may have contributed to an increase in blood viscosity leading to hyper-aggregation [57]. This is underlined by the finding of increased disaggregation threshold during RWR, necessary to separate the aggregates. Hyper-aggregation in the microvascular network has been found responsible for a decrease in arteriolar blood flow velocity and a reduction in perfused capillaries [58]. As a consequence, this reduces tissue perfusion and may decrease muscle strength, anaerobic power and endurance capacity of athletes [1,59–63].

The findings of the present study indicated that a rapid weight reduction, in contrast to gradual weight reduction, negatively influences RBC-NOS activation, NO production and thus hemorheological properties. Based on our previous findings [2] and the results presented herein it is concluded that RWR adversely affect microcirculatory perfusion, oxygen supply to the exercising muscles and as a consequence impair performance of TKD athletes. Thus, GWR should be preferred to achieve the desired weight prior to a competition to avoid impairments of hemorheological properties and NO signaling.

## Supporting Information

S1 Checklist TREND statement checklist. (PDF)
